# Evaluating the Efficacy and Safety of Repetitive Transcranial Magnetic Stimulation (rTMS) in Treating Prenatal Unipolar Depression: A Systematic Review

**DOI:** 10.1192/j.eurpsy.2025.1334

**Published:** 2025-08-26

**Authors:** D. O. O. Adedeji, M. Juhlin

**Affiliations:** 1 General Psychiatry, Helgeland Hospital, Mosjøen, Norway; 2 Neurobiology, Care Sciences, and Society, Karolinska Institutet; 3 General Psychiatry, Solna Sundbyberg Psykiatriska Mottagning, Stockholm, Sweden

## Abstract

**Introduction:**

Prenatal unipolar depression is a significant mental health challenge affecting a considerable number of pregnant women worldwide, with higher prevalence rates in developing regions. Due to the potential risks of antidepressant medications on fetal health, there is an increasing need for safe, non-pharmacological treatments. Repetitive transcranial magnetic stimulation (rTMS) has emerged as a promising neuromodulation therapy, offering a non-invasive approach to treating depression. This systematic review explores the efficacy and safety of rTMS in addressing prenatal depression, aiming to provide evidence for its potential as an alternative therapy for this vulnerable population.

**Objectives:**

The primary objective of this review was to assess the therapeutic effectiveness and safety profile of rTMS in treating unipolar depression among pregnant women. The secondary aim was to identify gaps in the current literature and provide recommendations for future research to strengthen clinical guidelines for rTMS use during pregnancy.

**Methods:**

A comprehensive search was conducted using the PubMed database without a time restriction, focusing on case reports, case series, uncontrolled studies, and randomized controlled trials that examined rTMS for prenatal depression. Inclusion criteria required studies to involve adult pregnant women diagnosed with major depressive disorder undergoing rTMS treatment. Data on depressive symptom improvements and any adverse effects on both mother and fetus were extracted for analysis.

**Results:**

Analysis of ten studies, including six case reports/series, three uncontrolled studies, and one randomized controlled trial, indicated that rTMS was well-tolerated by pregnant women and showed effectiveness in reducing depressive symptoms. Notably, no adverse effects were observed in fetal outcomes across these studies. Symptom improvement was significant, with high patient satisfaction reported, yet the limited sample sizes and variations in methodology underscore the need for more robust trials.

**Image:**

**Figure:**
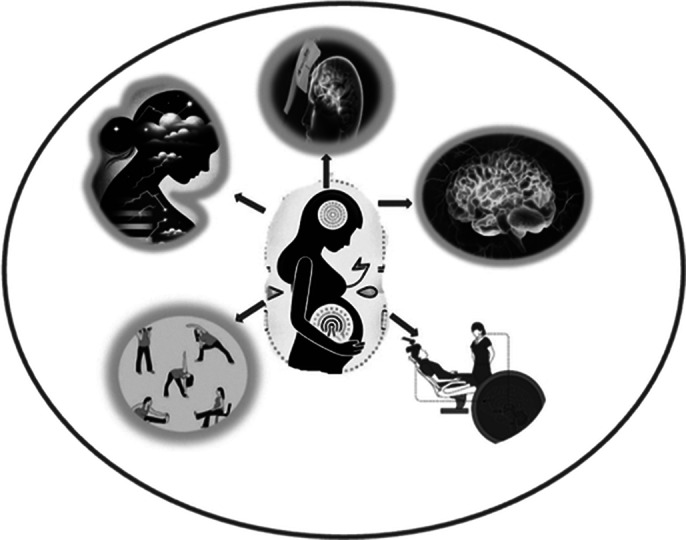

**Image 2:**

**Figure:**
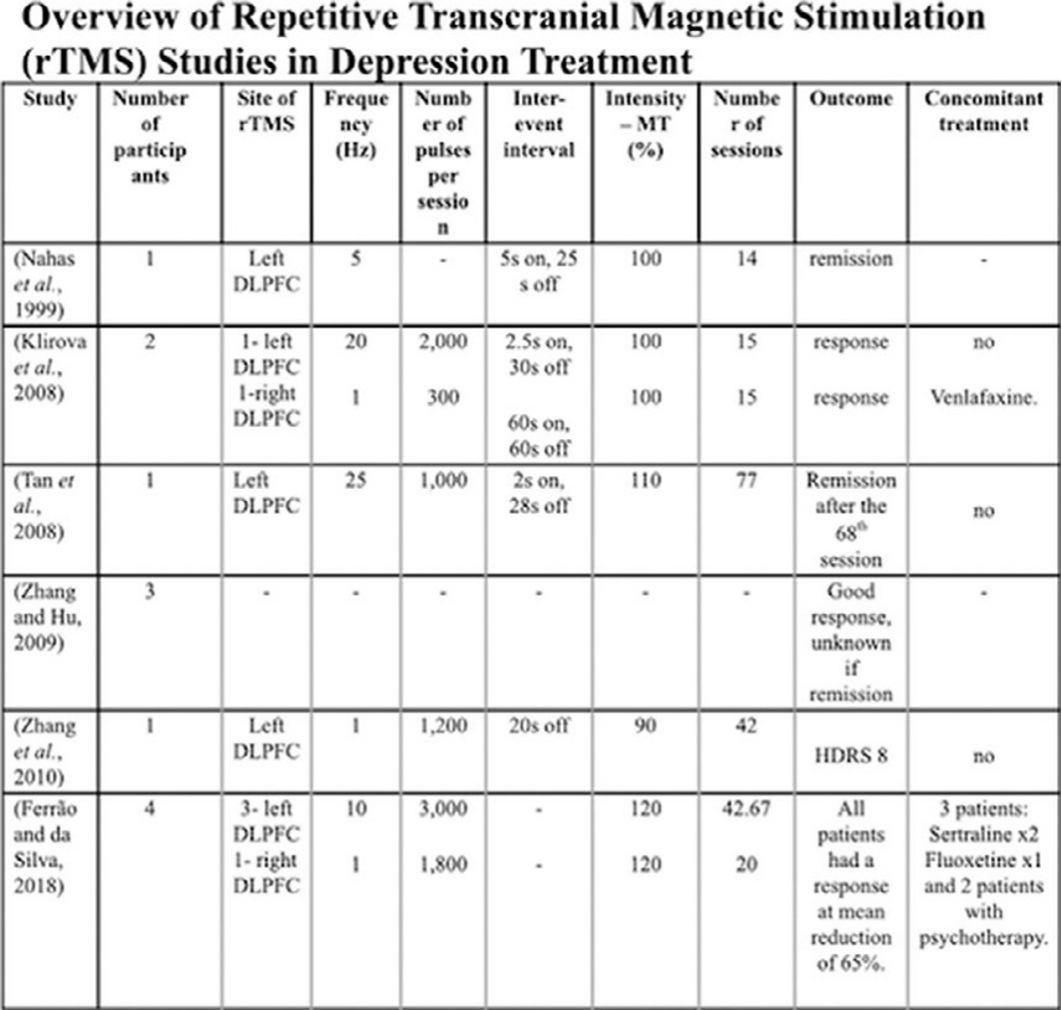

**Conclusions:**

The findings from this review suggest that rTMS is a promising, non-invasive alternative for managing prenatal depression with minimal fetal risk. However, due to the limited number of high-quality studies, further research is necessary to confirm these preliminary findings and establish standardized protocols for rTMS use in prenatal depression treatment. Expanding the evidence base will support the development of safe, effective treatment options tailored to the unique needs of expectant mothers facing depression.

**Disclosure of Interest:**

None Declared

